# Clinical outcomes in an adult patient with mannose phosphate isomerase-congenital disorder of glycosylation who discontinued mannose therapy

**DOI:** 10.1016/j.ymgmr.2020.100646

**Published:** 2020-09-07

**Authors:** Kinza Noman, Christian J. Hendriksz, Graham Radcliffe, Federico Roncaroli, Sulleman Moreea, Afifah Hussain, Karolina M. Stepien

**Affiliations:** a Medical School, University of Manchester, United Kingdom; b University of Pretoria, Steve Biko Academic Unit, Paediatrics and Child Health, Pretoria, South Africa; c Orthopaedic Department, Bradford Royal Infirmary, Bradford, United Kingdom; d Division of Neuroscience and Experimental Psychology, School of Biology, Medicine and Health, University of Manchester and Manchester Centre for Clinical Neuroscience, Salford Royal NHS Foundation Trust, United Kingdom; e Gastroenterology Department, Bradford Royal Infirmary, Bradford, United Kingdom; f Urgent Ambulatory Care, Bradford Royal Infirmary, Bradford, United Kingdom; g Adult Inherited Metabolic Diseases, Salford Royal NHS Foundation Trust, Salford M6 8HD, United Kingdom

**Keywords:** Mannose phosphate isomerase-congenital disorder of glycosylation, MPI-CDG, Phosphomannose isomerase, Adult, Clinical outcomes

## Abstract

The mannose phosphate isomerase-congenital disorder of glycosylation (MPI-CDG) is caused by phosphomannose isomerase deficiency. Clinical features include hyperinsulinaemic hypoglycaemia, protein losing enteropathy, hepatomegaly and hepatic fibrosis, digestive symptoms and coagulation abnormalities. The condition is treated with mannose supplementation. Long-term outcomes in adults are not well described. We present a case of an adult female patient who discontinued mannose therapy in her adolescence. In adulthood she developed gastrointestinal problems, chronic anaemia and osteophytes in her knees.

## 1 Introduction

The mannose phosphate isomerase-congenital disorder of glycosylation (MPI-CDG; OMIM 602579) is an autosomal recessive condition caused by deficiency of mannose phosphate isomerase (MPI) [1]. Other types of CDGs have features such as slow development, severe liver disease and neurological signs while MPI-CDG is characterised by severe liver involvement leading to cirrhosis. Hyperinsulinemic hypoglycaemia and abnormalities of coagulation, resulting in bleeding or a thrombotic tendency are other prominent features of the condition [2]. Vomiting, intractable diarrhoea and malnutrition occur in MPI-CDG patients due to protein-losing enteropathy, which is often associated with mild villous atrophy [3]. Patients affected by MPI-CDG show considerable phenotypic heterogeneity. Genetic analysis is always required to confirm the diagnosis.

MPI-CDG is treated with administration of oral mannose that bypasses the defective conversion of fructose 6-phosphate to mannose 6-phosphate [1,2,4]. Free mannose enters cells via a selective GLUT transporter present on the plasma membrane. Mannose is phosphorylated by hexokinase to produce mannose-6-phosphate, which serves as a common substrate for three competing enzymes, including MPI [5]. With mannose therapy the digestion improves and the risk of fatal complications such as gastrointestinal bleeding and the overall mortality are drastically reduced [1,4]. In contrast, mannose supplementary treatment does not reverse hepatic damage and as a result liver fibrosis evolves [1,4]. MPI-CDG is probably underdiagnosed and as a result, the evidence for the effectiveness of mannose treatment is limited.

We outline a 24-year follow-up of a female MPI-CDG patient, who was previously treated with mannose supplement in early childhood and adolescence, to emphasise clinical outcomes of the condition in adulthood.

## 2 Case

A South Asian female, homozygous for a novel D131N (c.391G > A) mutation in the *PM1* gene causing MPI-CDG [6,7], was born from consanguineous parents. At 6-months of age she presented with poor feeding and mild developmental delay. She was admitted to hospital several times with diarrhoea and vomiting. The patient was hypoglycaemic in these episodes; hypoglycaemia was attributed to hyperinsulinism. Serum albumin levels remained persistently low. At 9 months of age, congenital hepatic fibrosis was apparent. As part of investigations, agarose gel electrophoresis of serum transferrin showed a pattern specific to CDG Type 1 with abnormal glycosylation of transferrin. The patient had signs overlapping PMM2-CDG (a disease caused by reduced phosphomannomutase activity) such as thrombosis, inverted nipples, and fat pads on her chin and thighs [8].

Enzymatic assay of skin fibroblasts showed low MPI activity, which was causing incorrect glycosylation. At the time, this was a novel phenomenon, later termed MPI-CDG. No cognitive impairment was found. The patient was started on mannose which normalised her low anti-thrombin III and protein C in ten days; diarrhoea and vomiting gradually improved over the next 15 months. Hepatomegaly remained unchanged in this patient, though she had no significant health issues throughout her childhood and adolescence. The initial clinical presentation and her response to treatment had previously been detailed by Hendriksz and colleagues [7].

At the age of 16 years, prior to her transition from paediatric clinic, cardiology investigations detected no abnormalities. At the age of 18 years, mannose treatment was suspended due to the patient's poor compliance. Her liver function and renal function tests were normal. Coagulation parameters and iron studies were also satisfactory. By the age of 20 years, the patient had been hospitalised twice due to profound hypoglycaemia, which has now subsided. Her latest fasting glucose value was reported to be normal; her glucose was 4.3 mmol/L after prolonged fasting of 59 h [9].

The patient reported infrequent loose stools for years after stopping taking mannose. Since the age of 21 years, she was put under the care of gastroenterologists for her episodes of diarrhoea and abdominal pain. In a follow-up in the Adult Metabolic Clinic, attention was drawn to her BMI of 18.5 kg/m^2^ and risk of malnutrition, despite of sufficient caloric intake (approximately 2000 kcal). From the point of view that the patient has an underlying metabolic disorder, protein-losing enteropathy was suspected.

Thyroid and liver function tests, and HbA1c were normal in the patient. Haemoglobin concentration of 110 g/L and mean corpuscular volume (MCV) of 79.3 fl were in keeping with iron deficiency anaemia. The patient reported fatigue and tiredness despite repeated courses of iron supplements (Ferrous Sulphate 200 mg, 3 times a day) and previous iron infusions. Her menstrual cycle was regular and her hormonal profile (LH, FSH, oestradiol) was normal.

At the age of 22 years, haemoglobin had risen to 118 g/L whereas MCV and ferritin remained low, 78 fl and 11 μg/L respectively. Furthermore, vitamin D was insufficient at 42 nmol/L. All other biochemical tests including plasma glucose, total cholesterol, Hba1c, and protein S were satisfactory.

The patient also tested positive twice for anti-transglutaminase antibodies confirming that chronic anaemia, gastritis and low weight could be attributed to coeliac disease. Her duodenal biopsy was consistent with the condition as it showed mild T-lymphocyte infiltrates in the epithelium with focal shortening of villi, though no overt villous atrophy was found. Signs and symptoms of gluten intolerance at onset were very mild and the condition was excluded in her childhood [7]. The patient currently adheres to a gluten-free diet; her fatigue and body weight improved. She refused to be re-established on mannose.

As the condition may present with liver fibrosis at any stage of life, liver function tests and AFP continued to be monitored and were normal. On examination there were no features of chronic liver disease. USS and CT scan of her liver and spleen were normal. Close monitoring of her clotting profile is prudent. At the age of 22 years, her protein C was low at 65 u/dl (70–140) and free protein S was 77 u/dl (53–123), the latter being normal but markedly reduced from a previous titre of 96 u/dl. Low protein C increases the risk of thrombosis. Her prolonged prothrombin time of 15.5 s increases her risk of haemorrhage. Coagulation abnormalities are expected in MPI-CDG patients [4].

**Fig. 1:**
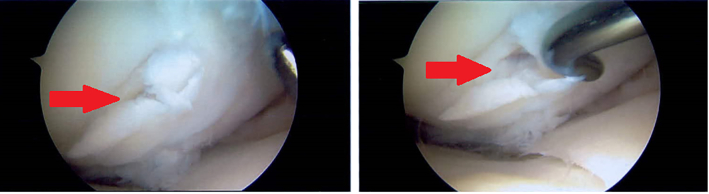
Arthroscopic images of the medial femoral condyle of the right knee demonstrating an unstable full thickness osteochondral flap lesion.

**Fig. 2:**
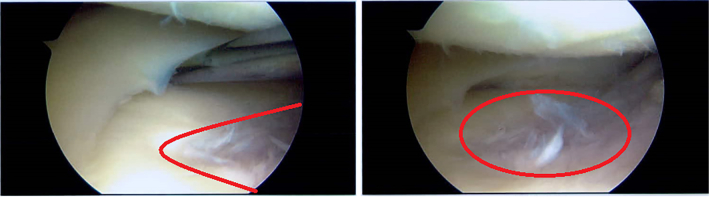
Arthroscopic images of the lateral compartment of the right knee demonstrating more superficial chondrial fibrilation and normal meniscal tissue. Abnormality outlined in red.

At the age of 20 years, due to persistent knee pain irresponsive to analgesia, the patient underwent staged bilateral knee arthroscopies under general anaesthetic. She was prepared for theatre with a Polycal drink (Emergency regimen) two hours prior to the induction of anaesthetic and blood glucose was monitored very closely throughout and after the anaesthetics. Oral Polycal and intravenous 50% Dextrose infusions were prescribed as required in the peri-operative period. The progress of and recovery from general anaesthesia was unremarkable on both occasions. The findings at arthroscopic surgery were of extensive osteochondritis dissecans affecting both femoral condyles of the left knee and predominantly the medial femoral condyle of the right knee. There were full thickness unstable osteochondal flaps and significant chondral debris in both knees (Fig. 1, Fig. 2).

**Fig. 3:**
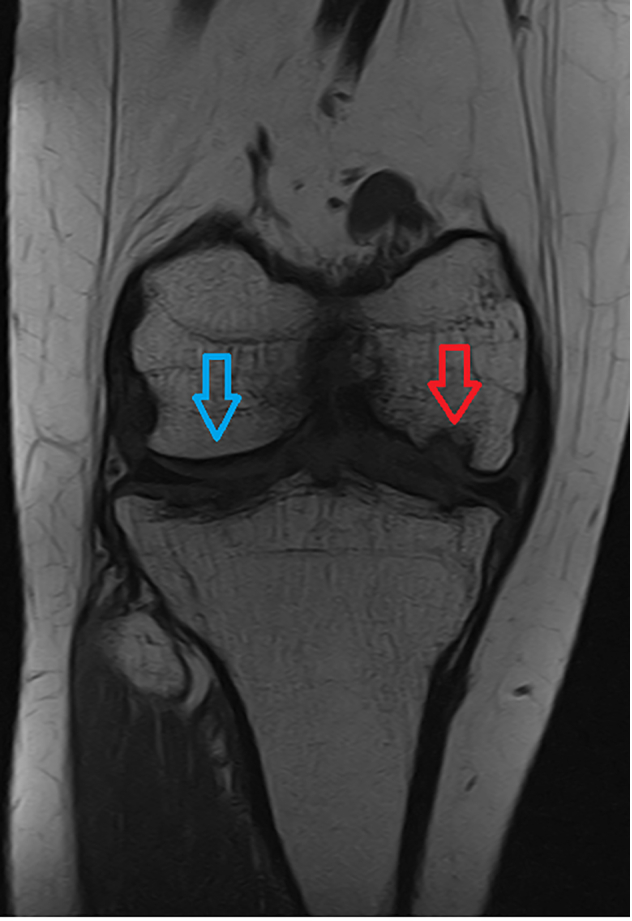
T1 weighted coronal MRI image showing extensive defect of medial femoral condyle in comparison with relatively normal lateral femoral condyle. Red arrow indicates medial lesion. Blue arrow indicates noral lateral femoral condyle.

Surgery gave good relief of her pain on the left knee but symptoms were persistent on the right side. An MRI of her right knee identified progressive disintegration of the chondral surface of the knee and the formation of further unstable osteochondral flaps (Fig. 3).

She underwent a further right knee arthroscopy at the age of 23 years. General anaesthetic was managed similarly and was unremarkable. The findings were of progression and extension of the osteochondritis and grade III degenerate change. Histopathological review of the tissue sample showed a small fragment of normal appearing cartilage. The patient reports significant benefit from the surgeries and her knee pain is controlled with codeine and paracetamol as required at the time of writing.

Our patient has normal IQ and she has completed a course in nursing.

## 3 Discussion

MPI-CDG has few clinical findings as opposed to other CDG types where specific features like psychomotor retardation, cerebellar hypoplasia, strabismus and lipocutaneous abnormalities are evident [8]. Major hallmarks of MPI-CDG are protein losing enteropathy and hepatic fibrosis [10]. The correction of the transferrin profile in childhood demonstrated that mannose was successful in treating our patient and demonstrated that impaired synthesis in the liver was reversed [1,4]. Patients without any liver disease [8] are those where complete correction of the glycosylation profile was observed during mannose therapy [1]. In view of the rarity of adult MPI-CDG cases [11,12], the benefits of taking mannose long-term are not known. Oral mannose supplementation at the dose of 0.1–0.17 g mannose/kg (given orally six times a day) remains a treatment option for MPI-CDG [4] and has been previously shown to correct postprandial plasma glucose, insulin and C-peptide levels, decreased aminotransferase activity, and increased coagulation factor levels [8,[10], [11], [12]]. Biochemical and haematological parameters improved in our patient as well as in published examples in parallel to the administration of mannose [1,8]. Therefore, patients who were established on the mannose therapy in childhood and who do not experience significant adverse effects, are encouraged to continue on the therapy life-long.

Mannose supplement is generally well tolerated by patients [8,[11], [12], [13]]. It was suggested that heparin could be used as an alternative to mannose in treating protein-losing enteropathy, particularly in cases with protein-enteropathy is non-responsive to mannose therapy [1]. Another 28-year old woman, who developed progressive liver fibrosis despite mannose and heparin therapy, showed successful resolution of her symptoms in a 2-year follow-up after liver transplantation [14]. New therapies for CDG are currently under development; pharmacological chaperone therapy, antisense therapy or gene therapy [15].

Our patient developed hypoglycaemia, relapsing gastrointestinal complications, and chronic anaemia in her adulthood. These features possible signify the progression of MPI-CDG; this is not clear as the clinical course of the condition in adulthood is not fully understood due to its rareness. Our patient represents one of the oldest living patients affected with this condition. Another 32-year old female was incidentally found to have MPI deficiency, but this individual remained asymptomatic to this day [16]. Other cases of 33-year-old [11] and 35-year-old females [12] describe their long-term clinical outcomes while on the therapy with mannose.

Another explanation for the patient's symptoms is coeliac disease, which, similarly to MPI-CDG, is directly related to protein-losing enteropathy and iron deficiency. In undiagnosed patients, ongoing experiences of poor feeding, diarrhoea, and anaemia are common.

In addition, this is the first case of MPI-CDG presenting with enchondromatosis (Ollier's disease). Ollier's disease is a low prevalence condition with an incidence of 1:100,000. Joint diseases have been previously described in *N*-linked disorders of glycosylation with thorax shortening and scoliosis documented in CDGs [17] while Ollier's disease in our patient likely represents a coincidental condition.

In conclusion, this case emphasises that the long-term efficacy of mannose supplementation is unknown and there are several uncertainties with regards to adult presentation of MPI-CDG. It is advisable that future patients that present similarly, i.e. with gastrointestinal complications, low weight and iron-deficiency anaemia, should be investigated for coexisting pathologies, which may not be direct complications of the underlying metabolic condition, but may aggravate their symptoms.

## Funding

N/A.

## Patient consent

The patient consented to this study.

## Guarantor

KMS.

## Declaration of Competing Interest

The authors have no conflict of interest.
